# Molecular basis of high temperature-induced bolting in lettuce revealed by multi-omics analysis

**DOI:** 10.1186/s12864-022-08814-z

**Published:** 2022-08-12

**Authors:** Jinghong Hao, Junwei Yang, Xiaofeng Liu, Gaoyang Pan, Yunfeng Li, Xiaolan Zhang, Yingyan Han, Shuangxi Fan, Zhaoyang Zhou

**Affiliations:** 1 Beijing Key Laboratory of New Technology in Agricultural Application, National Demonstration Center for Experimental Plant Production Education, Plant Science and Technology College, Beijing University of Agriculture, Beijing, China; 2 College of Horticulture, China Agricultural University, Beijing, 100193 China; 3 State Key Laboratories of Agrobiotechnology, Beijing Key Laboratory of Growth and Developmental Regulation for Protected Vegetable Crops, College of Horticulture, China Agricultural University, Beijing, 100193 China

**Keywords:** Lettuce (*Lactuca sativa* L.), High temperature, Bolting, Multi-omics

## Abstract

**Background:**

High temperature induces early bolting in lettuce (*Lactuca sativa* L.), which affects both quality and production. However, the molecular mechanism underlying high temperature-induced bolting is still limited.

**Results:**

We performed systematical analysis of morphology, transcriptome, miRNAs and methylome in lettuce under high temperature treatment. Through a comparison of RNA-Seq data between the control and the high temperature treated lettuces at different time points totally identified 2944 up-regulated genes and 2203 down-regulated genes, which cover three floral pathways including photoperiod, age and gibberellin (GA) pathways. Genome wide analysis of miRNAs and methylome during high temperature treatment indicated miRNAs and DNA methylation might play a role controlling gene expression during high temperature-induced bolting. miRNA targets included some protein kinase family proteins, which potentially play crucial roles in this process.

**Conclusions:**

Together, our results propose a possible regulation network involved in high temperature-induced bolting.

**Supplementary Information:**

The online version contains supplementary material available at 10.1186/s12864-022-08814-z.

## Introduction

Plants undergo different developmental stages in their life cycle [1]. Among them, the transition from vegetative to reproductive growth is a crucial step. In this process, the shoot apical meristem (SAM) plays a vital role. During vegetative stage, the SAM produces leaves from the peripheral zone. Upon translation, the SAM elongates and converts into the inflorescence meristem (IM), generating flowers [2].

This floral transition is tightly controlled by developmental, hormonal and environmental signals. Among them, the photoperiod and temperature are the most important environmental cues [3]. In *Arabidopsis*, previous studies have shown that six major genetic pathways, including photoperiod, ambient temperature, age, gibberellin (GA), autonomous, and vernalization pathways, control this transition [4]. The flowering locus T (FT) and suppressor of overexpression of constans 1 (SOC1/AGL20) are among the most important floral pathway integrators that control the transition to flowering [5]. FT acts as a long-distance signal molecular, moving from the leaves to the apical meristem through the phloem and promoting flowering at the SAM [6, 7]. Previous studies have shown that many transcription factors are involved in the transcription regulation of FT. The photoperiod-associated factors constans (CO) and phytochrome-interacting factor 4 (PIF4) activate *FT* expression in response to long-day (LD) conditions and increasing temperature [8, 9], while vernalization-dependent gene flowering locus C (FLC) and APETALA 2 (AP2) -like genes repress *FT* expression [10, 11]. The transcription level of another floral integrator gene *SOC1* is mainly regulated by CO and FLC [12]. CO promotes *SOC1* expression mainly through FT, while FLC represses *SOC1* transcript by binding to its promoter. Subsequently, FT activates the downstream floral meristem identity genes *AP1* and its paralog *CAULIFLOWER* (*CAL*) [13], and SOC1 activates *LEAFY* (*LFY*), leading to floral initiation [14].

Although the genetic network of flower transition is well-studied in *Arabidopsis*, this knowledge in most crops is still lacking. Lettuce (*Lactuca sativa*), belonging to Compositeae family, is an important leafy vegetable worldwide [15]. Cool temperature is suitable for its cultivation, and high temperature induces its bolting (rapid stem elongation), which is irreversible and seriously affects the quality of harvestable crops and flavor [16]. So, studying the mechanism of heat-induced bolting is important for lettuce breeding. Previous studies showed that knockdown the homolog of the *Arabidopsis SOC1* or *FT* in lettuce led to a bolting delay with reduced *LFY* and *AP1* expression and insensitivity to high temperature [17, 18], indicating that *FT*, *SOC1*, and their downstream genes *AP1* and *LFY* likely play important roles in lettuce floral transition. In addition, GA and related genes were reported to be associated with bolting in response to high temperatures [19–21]. These studies suggested that the function of these central genes in floral translation seems to be conserved between lettuce and *Arabidopsis*, but FT and SOC1 integrate which floral pathways to regulate high temperature-induced bolting in lettuce remain largely unknown.

To further investigate the relevant signaling pathway involved flowering transition in lettuce, RNA-Seq, miRNAs and DNA methylome were performed in lettuce tips during high temperature treatment. RNA-Seq data showed that three floral pathways, including photoperiod, age and gibberellin (GA), are potential involved in high temperature-induced bolting. Moreover, epigenome analysis indicated that miRNAs and DNA methylation may be also involved in bolting by regulating floral pathways indirectly. Together, our study provided new molecular insights into the mechanism of high temperature-induced bolting in lettuce.

## Results

### The effect of high temperature on lettuce bolting

**Fig. 1:**
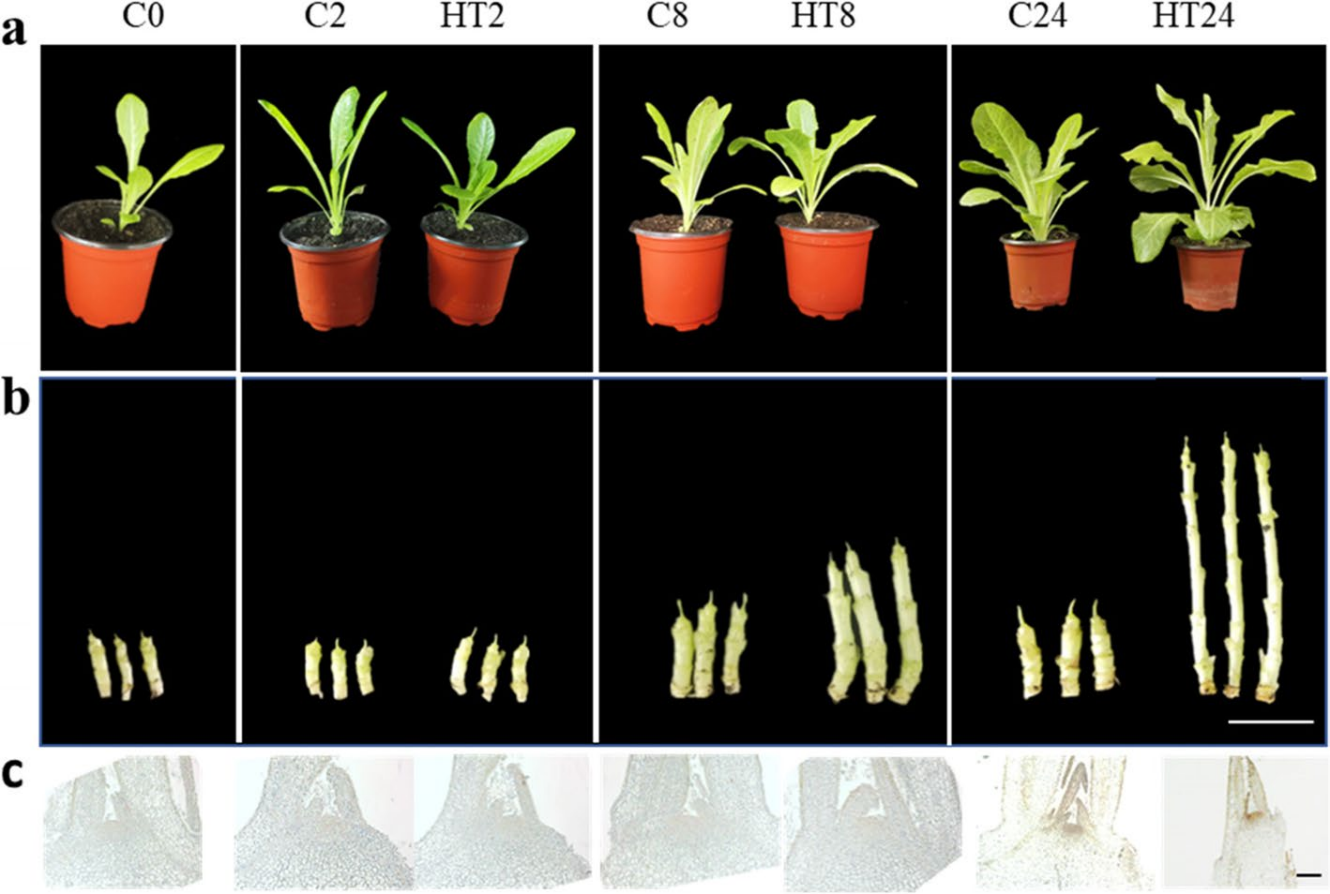
High-temperature induced bolting in lettuce. **a** Morphology of lettuce seedlings grown under suitable temperature (control, C) and high temperature (HT) treatment. The photographs are representative for three independent experiments; **b** Changes in stem length of the control and high temperature-treated lettuce seedlings. Bar = 2 cm; **c** Histological sections showing shoot apical meristem of lettuces grown under control and HT treatment. Bar = 500 μm

To study high temperature-induced floral transition in lettuce, the seedlings with seven leaves were transferred to the plant incubators with 14 h light at 33 °C and 10 h darkness at 25 °C, while the control was cultured at 20 °C on the day and 13 °C at night. We observed that the high temperature treatment could induce a longer stem compared to the control group at the 8th day (C8) (Fig. 1a, b). After 24 day treatment, the lettuce stem has elongated to approximately 7 cm (Fig. 1a). To further analyze the morphological changes of lettuce stem tips, histological sections were performed to observe the morphological features at different time points. As shown in Fig. 1, compared to control at 24th day (C24), the SAM of the lettuce treated by high temperature at 24th day (HT24) is becoming from flat to domed, marking the transition from vegetative to reproductive growth with elongated SAM (Fig. 1c). So, the lettuce at C0, C2, C8, C24, HT2, HT8 and HT24 were selected for further study.

### Transcriptome reprogramming during high temperature-induced bolting

**Fig. 2:**
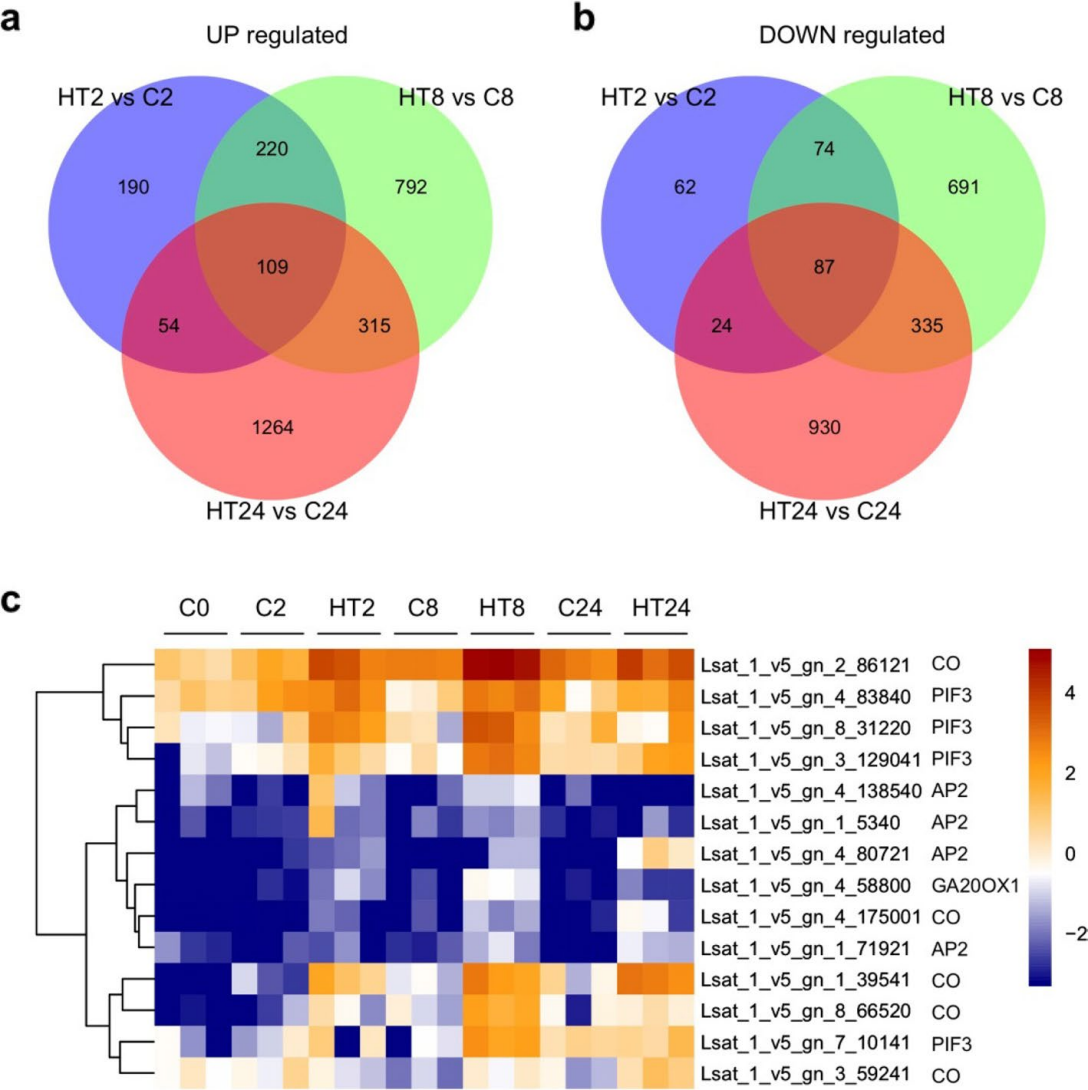
Differentiallly expressed genes (DEGs) in the lettuce tips in pairwise comparisons HTs vs Cs. **a-b** Venn diagram showing the number of up- and down-regulated DEGs; (**c**) Heatmaps showing the expression profile of genes involved in flower transition pathway. The brown color indicated higher expression while blue indicated lower expression

To explore which pathways were involved in high temperature induced bolting in lettuce, we performed RNA-Seq of the same control and high-temperature treated lettuces as in morphological analysis. Hiseq-PE150 sequencing produced about 6 Gb data for sample. The principal component analysis (PCA) revealed homogeneity between replicates at each time point (Fig. S1). We identified 2944 up-regulated differentially expressed genes (DEGs) and 2203 down-regulated DEGs totally by comparison between the control and the high temperature treated lettuces (Fig. 2a-b).

Gene Ontology (GO) enrichment analysis was performed at different time points to probe the pathway involved in high temperature-induced bolting. For those up-regulated DEGs under high temperature treatment, the significant enrichment pathways included ‘transcription factors and regulators’ and ‘trans-membrane signaling receptor’ (Fig. S2-S4). For those down-DEGs under high temperature treatment, the ‘heat shock protein binding’ was shown to be enriched significantly (Fig. S2-S4). Moreover, the Kyoto Encyclopedia of Genes and Genomes (KEGG) enrichment analysis showed that the plant hormone signal transduction is the significant enrichment pathway after high temperature treatment for 8 days (Fig. S2-S4). It is noteworthy that those pathways may be highly related to bolting in lettuce.

### Dynamic expression of genes related to flower transition during high temperature-induced bolting

To study the DGE expression pattern, these DEGs that may be involved in the flowering pathway were selected for analysis. The result showed that the expression levels of most these DGEs were induced by high temperature treatment (Fig. S5). Previous studies reported that the floral pathway integrators *SOC1* and *FT* play important roles in lettuce bolting [17, 18], we first analyzed the expression levels of *SOC1* and *FT* based on the RNA-Seq data. Unfortunately, the expression of *FT* (Lsat_1_v5_gn_2_17881) was too low to be detected. The expression of *SOC1* (Lsat_1_v5_gn_9_53801) was began to up-regulated after high temperature treatment at 24th day (Fig. S6a), indicating that *SOC1* may play an important role in lettuce bolting. These results prompted us to further investigate which floral pathway involved in flower transition regulation from the RNA-Seq data. Among these genes, most of them are *COs* and *PIFs*, which are involved in photoperiod pathways. Meanwhile, the gene *GA 20-OXIDASE 1* (*GA20OX1*) responsible for GA biosynthesis was significantly up-regulated in high temperature-treated lettuce at 8th day (Fig. 2c). Moreover, some *AP2-like* genes in age pathway were down-regulated after high temperature treatment at 24th day (Fig. 2). Apart from them, vernalization is also a major determinant of flowering time, which leads to down regulation of *FLC* to promote flower [3]. To study whether vernalization pathway is involved in high temperature-induced flowering in lettuce, we analyzed the expression levels of vernalization pathway genes *VIN3* and *FLC* after high temperature treatment. The expression level of *VIN3* was not changed, which *FLC* was down-regulated after high temperature treatment (Fig. S6), indicating that vernalization pathway may be also required for heat induced flowering in lettuce. Taken together, these data suggested that multiple pathways may be involved in the regulation of high temperature-induced bolting in lettuce.

### Dynamic changes in microRNAs during high temperature-induced bolting

**Fig. 3:**
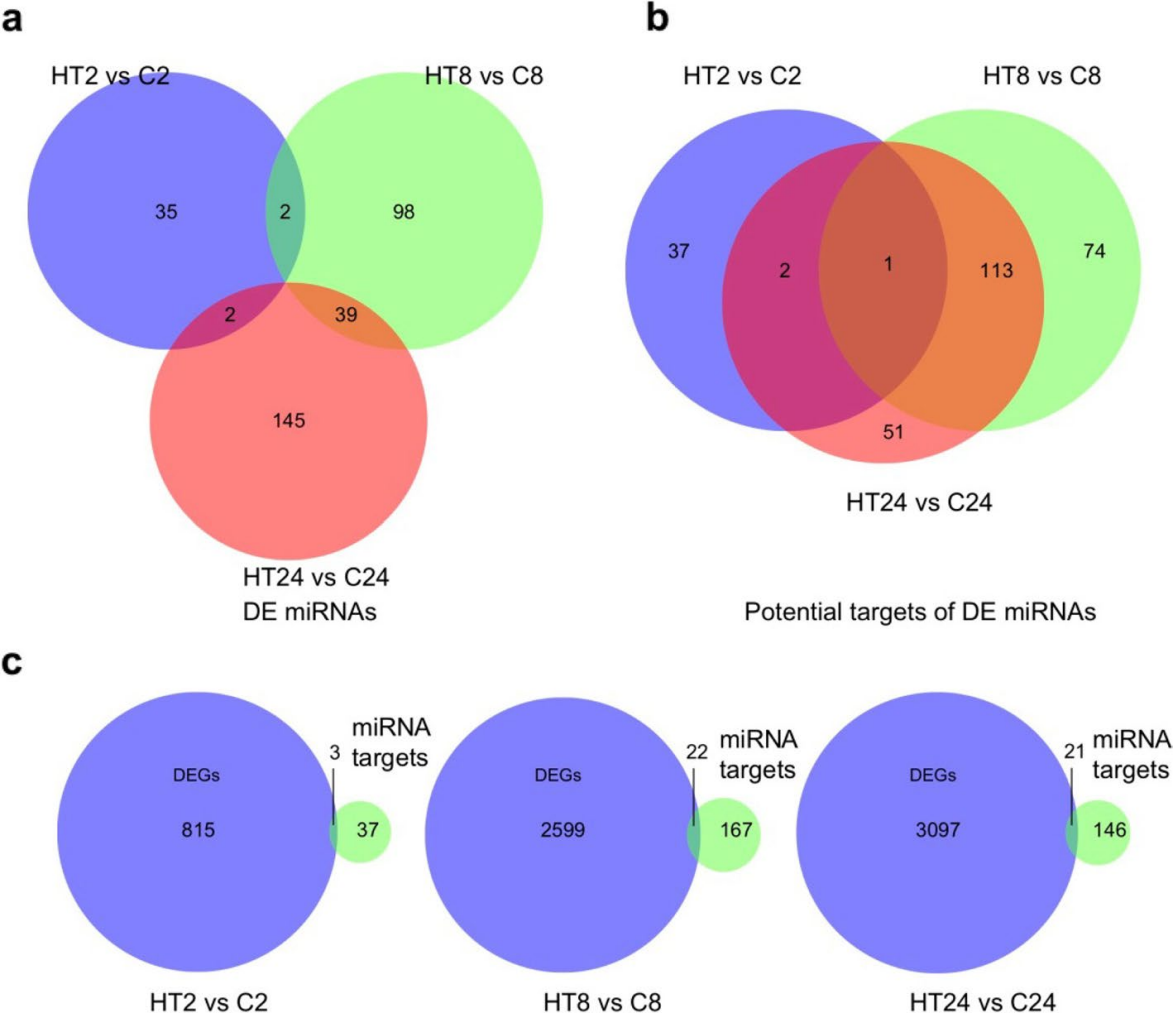
Differentially expressed (DE) miRNAs in lettuce by comparing HT2 vs C2, HT8 vs C8 and HT24 vs C24. **a** The number of up- and down-regulated miRNAs shown by venn diagrams; **b** The number of anticorrelated targets of DE miRNAs shown by venn diagrams; **c** Overlap between DEGs and miRNAs target genes upon high temperature treatment

MicroRNAs (miRNA) play important roles in gene expression regulation by targeting mRNAs [22]. To identify whether miRNAs potentially regulate high temperature-triggered bolting, small non-coding RNA sequencing was performed in different lettuce samples (i.e., C0, C2, C8, C24, HT2, HT8 and HT24). In total, 5295 unique miRNAs were identified in lettuce. As expected, the expression levels of some miRNAs were increased gradually in lettuce with increasing high temperature treatment (Fig. S7). To study the potential role of miRNAs in regulating gene expression during high temperature induced bolting, the differential analysis was performed in HT2 vs C2, HT8 vs C8 and HT24 vs C24 and identified 39, 139 and 186 differentially expressed (DE) miRNAs, respectively (Fig. 3a, Table S1). These DE miRNAs potentially targeted 40, 188 and 167 coding genes in HT2 vs C2, HT8 vs C8 and HT24 vs C24, respectively (Fig. 3b, Table S1). Among them, 3, 22 and 21 genes were overlapped between the DEGs and these coding genes potentially targeted by miRNAs during high temperature treatment at 2, 8 and 24 day, respectively (Fig. 3c, Table S2–4), suggesting that the expression of some DEGs were potentially affected by miRNAs (Fig. 3c), although no gene was reported to be involved in the known floral pathway. Among these potentially targeted coding genes, there are some kinase protein family proteins, which may be involved in bolting regulation (Fig. 3c, Table S2–4). These results suggested that the involvements of miRNAs during high temperature treatment possibly affect coding gene expression and bolting in lettuce.

### DNA methylation patterns during high temperature-induced bolting

**Fig. 4:**
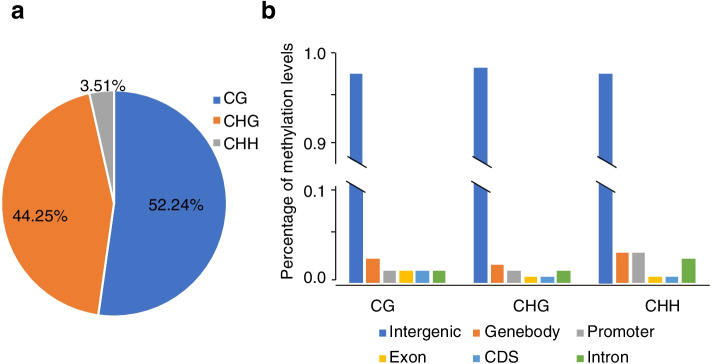
The methylome of lettuce. **a** Relative proportions of mCs in three sequence contexts (CG, CHG and CHH) in lettuce; **b** Percentage of methylation levels of intergenic, genebody, promoter, exon, intron and CDS sequences in normal samples

To investigate the overall methylation patterns of lettuce response to high temperature, control and high temperature treated lettuces at different time points were selected for methylation analysis. Each sample produced about 25 Gb data. In the C0 sample, methylcytosine occurred at CHH sites (3.51%), followed by CHG (44.25%) and CG (52.24%) (Fig. 4a). To further explore DNA methylation distribution in different genomic regions, we analyzed the methylation profiles with genes and their flanking sequences. Our results showed that methylation occurs mostly at intergenic regions (Fig. 4b). Moreover, methylation at CGs, CHGs and CHHs within protein-encoding genes exhibited different distribution across sequence contexts. CG and CHG methylation primarily occurred in the genebodies, while methylation at CHHs preferentially occurred in both genebodies and promoters (Fig. 4b).

**Fig. 5:**
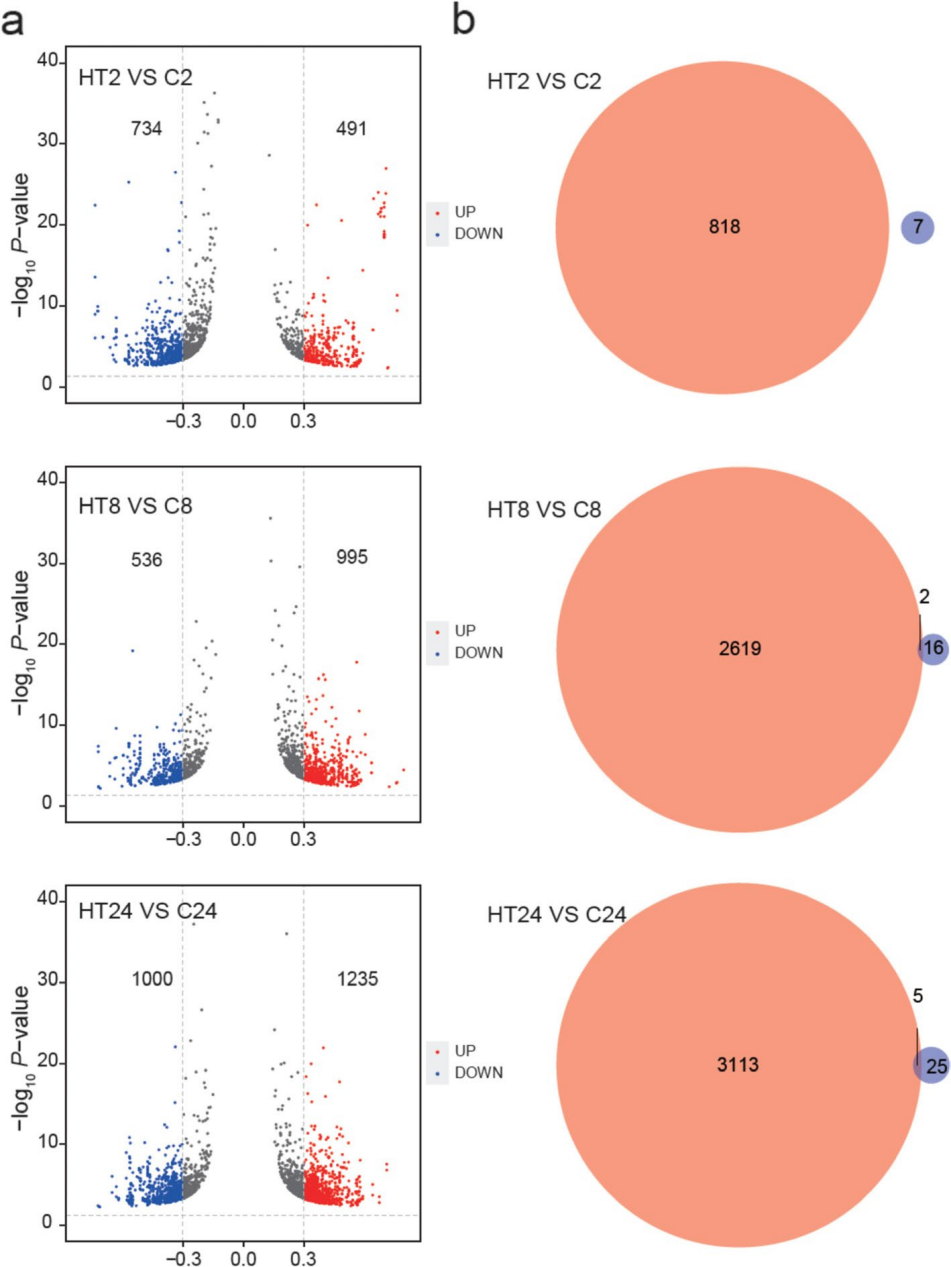
Dynamic methylation changes in lettuce during high temperature treatment. **a** Differentially methylated locis (DMLs) in lettuce tips during high temperature treatment; **b** Overlap between Differentially methylated region (DMR)-associated genes and DEGs under high temperature treatment in lettuce

To examine the DNA methylation patterns in high temperature-induced bolting, we analyzed the changes of DNA methylation after high temperature treatment. It can be seen that with the increase of treatment time, the number of differentially methylated region was increased significantly (Fig. S8). To further explore whether any role of DNA methylation in high temperature induced-lettuce bolting, we compared the DNA methylomes in HT2 vs C2, HT8 vs C8 and HT24 vs C24 to identify the differentially methylated locis (DMLs). There are 1225, 1531, and 2235 DMLs, respectively (Fig. 5a). In HT2 vs C2, 67 DMLs were located in gene bodies and 44 DMLs in gene promoters. In HT8 vs C8, there are 183 DMLs in gene bodies and 19 DMLs in gene promoters. In HT24 vs C24, 358 and 76 DMLs fell into gene bodies and promoters, respectively (Table S5). We further identified differentially methylated regions (DMRs) using the R packages “DSS” and these DMRs were associated with 7, 16 and 25 protein-coding genes at 2d, 8d and 24d, respectively (Fig. 5b, Table S6). However, only 0, 2 and 5 genes were overlapped between the DMR-associated genes and DEGs at 2d, 8d and 24d during high temperature treatment (Fig. 5b, Table S7–8), indicating a potential role of DNA methylation in regulating gene expression during high temperature-induced bolting.

## Discussion

In this study, we performed the transcriptome and epigenome analysis to study the molecular mechanism of high temperature-induced bolting. Our results unravelled that multiple floral pathways are involved in this process.

It is well known that there are six major genetic pathways controlling flower transition in *Arabidopsis* and these flowering signals converge at two regulators, FT and SOC1 [4]. Previous studies showed that high temperature treatment could promote *LsFT* and *LsSOC1* expression in lettuce. *LsFT* or *LsSOC1* knockdown by RNA interference led to insensitive to high temperature and late bolting phenotype [17, 18]. But which floral pathways are involved in *LsFT* and *LsSOC1* expression regulation during high temperature-induced bolting is lacking. RNAseq analysis performed in our study revealed that thousands of genes were differentially expressed after high temperature treatment. Among them, most of the flowering time genes are *PIF* and *CO* genes. Consistently, recent study also showed that *CO LIKE-9* (*COL9*) was identified as a candidate affecting flowering and bolting in lettuce by utilizing a lettuce mapping population [23], indicating that photoperiod/clock pathway may be a major pathway for high temperature-induced bolting. Consistent with previous study that high temperature treatment results in increased expression level of *LsGA2ox1*, *LsGA3ox1* with corresponding enhanced GA8 and GA1 endogenous levels [19, 21], our results showed the gene *GA20OX1* responsible for GA biosynthesis was significantly up-regulated after high temperature treatment for 8 days. Moreover, some *AP2*-like genes in age pathway were down-regulated after high temperature treatment (Fig. 2), indicating that hormonal and age pathways are also involved in high temperature-induced bolting.

Given that epigenome reprogramming plays an important role in gene expression regulation, genome-wide analysis of miRNAs and DNA methylation was performed in this study. We found that a few of DEGs were potentially targeted by DE miRNAs and/or associated with DMRs. Our results showed that about 6% DEGs including some protein kinase family proteins were potential targets of miRNAs during high temperature-induced bolting, although none is known to play a role in at first glance. Previous studies have demonstrated that some protein kinase family proteins, such as CLAVATA1 (CLV1) and CLV2, regulate flower time [24, 25]. So, it will be interesting to study whether these protein kinases are involved in high temperature-induced bolting in the future.

## Conclusions

Together, our study provided new molecular insights into the mechanism of high temperature-induced bolting by multi-omics analysis. To characterize the functions of the candidate genes will promote the understanding this process and facilitate lettuce breeding in the future.

## Methods

### Plant materials and growth conditions

The heat-sensitive *Lactuca sativa* L. cultivar GB-30, an easy bolting variety, was used in this study. The lettuce was grown in greenhouse of Beijing University of Agriculture Experimental Station with 14 h light at 20 °C/10 h darkness at 13 °C. When the seedlings have seven true leaves, they were randomly divided into two groups and treated with different temperatures: 33 °C as the treatment and 25 °C as the control. These seedlings were treated for 24 days.

### RNA-Seq analysis and enrichment analysis

RNA-Seq was performed by novogene using the Hiseq-PE150 platform. Tips of the lettuce at four different time points (0d, 2d, 8d, 24d) under normal and high temperature treatments were collected separately in three biological replicates and subjected to RNA-Seq analysis. Differentially expressed genes (DEGs) were identified based on lettuce RNA-Seq data from different treatments. All reads were mapped to the lettuce reference genome with default parameters using HISAT2 software (https://daehwankimlab.github.io/hisat2, version 2.1.0), and assemble transcripts by StringTie (http://ccb.jhu.edu/software/stringtie/, version 2.0.3) from the read alignment data. DEGs were identified through the package ‘DEseq2’ in R software (version 3.6.0) for RNA-Seq data from two treatments with the cutoff: P < = 0.05 and fold change > = 2. The expression patterns of those candidate DEGs among the HT2 vs C0, HT8 vs HT2, and HT24 vs HT8 were displayed according their FPKM using R package ‘pheatmap’. Gene ontology (GO) and Kyoto Encyclopedia of Genes and Genomes (KEGG) enrichment analyses were conducted for both the up-regulated and down-regulated genes using the software “TopGO” and “clusterProfiler” [26], respectively.

### Identification of miRNAs and their targets

smallRNA-Seq (sRNA-Seq) was performed by novogene using the Nova-SE50 platform. The lettuce miRNAs were predicted by miR-PREFeR (miRNA PREdiction From small RNA-Seq data) (https://github.com/hangelwen/miR-PREFeR.git, version 0.23.0) default pipline. Differentially expressed (DE) miRNAs between the control and high temperature treated lettuces were identified using the package ‘DEseq2’, and their patterns were showed using R package ‘pheatmap’. Then, targets of those differentially expressed (DE) miRNAs between the control and high temperature treated lettuces were predicted by TargetFinder (https://github.com/carringtonlab/TargetFinder.git).

### Methylation analysis

BS-seq was performed by novogene using the Hiseq-PE150 platform. For methylation, clean reads of BS-seq data were mapped using Bismark software (https://github.com/FelixKrueger/Bismark) against the lettuce reference genome, which was firstly transformed to bisulfite-converted version and then index using bowtie 2 (http://bowtie-bio.sourceforge.net/bowtie2/index.shtml, version 2.4.2). Differentially methylated regions (DMRs) were identified using the R packages “DSS” (Dispersion Shrinkage for Sequencing data), and the methylated patterns among the HT2 vs C0, HT8 vs C0, and HT24 vs C0 were displayed by circle plot using the shinyCircos [27]. The genes in the DMRs were considered to be methylation related genes for further analysis.

## Supplementary Information

**Additional file 1: Supplementary Fig. 1.** Principal component analysis (PCA) of control and high temperature treated samples at different time points. PCA of the 21 RNA-Seq datasets showed 7 distinct groups. RNA-Seq data related to control at 0 day (C0), control at 2 day (C2), control at 8 day (C8), control at 24 day (C24), 2 days post high temperature treatment (HT2), 8 days post high temperature treatment (HT8) and 24 days post high temperature treatment (HT24). **Supplementary Fig. 2.** GO and KEGG analysis of differentially expressed genes (DEGs) in lettuce tips between HT2 and C2. (**a**-**b**) Scatter diagram of GO enrichments of up (**a**)- and down (**b**)-DEGs between HT2 and C2. (**c**-**d**) Scatter diagram of KEGG enrichments of up (**c**)- and down (**d**)-regulated DEGs between HT2 and C2. Gene ratio is the significant DEG number to the background number in a specific pathway. The dot color represents -log_10_(*P*), and a higher value indicates greater pathway enrichment. **Supplementary Fig. 3.** GO and KEGG analysis of DEGs in lettuce tips between HT8 and C8. Scatter diagram of pathway enrichments of up (**a, c**)- and down (**b, d**)-regulated DEGs after high temperature treatment at 8 day. Gene ratio is the significant DEG number to the background number in a specific pathway. The dot color represents –log_10_(*P*), and the red dot color indicates greater pathway enrichment. **Supplementary Fig. 4.** GO and KEGG analysis of differentially expressed genes (DEGs) in lettuce tips between HT24 and C24. Scatter diagram of pathway enrichments of up (**a, c**)- and down (**b, d**)-regulated DEGs post high temperature treatment at 24 day. Gene ratio is the significant DEG number to the background number in a specific pathway. The red dot color indicates greater pathway enrichment. **Supplementary Fig. 5.** Gene expression pattern and functional category over the time course during high temperature treatment. The brown color indicated higher expression while blue indicated lower expression. **Supplementary Fig. 6.** The expression level of *SOC1*, *FT*, *VIN3* and *FLC* based on the RNA-Seq data. **Supplementary Fig. 7.** The expression pattern of miRNAs over the time course during high temperature treatment in lettuce. The brown color indicated higher expression, while blue indicated lower expression. **Supplementary Fig. 8.** The distribution patterns of differentially methylated regions (DMRs) on chromosomes in lettuce.

**Additional file 2. Table S1. **DE miRNA target genes.

**Additional file 3. Table S2. **Overlap between DEGs and DE miRNA targets during high temperature treatment at 2 day

**Additional file 4. **Overlap between DEGs and DE miRNA targets during high temperature treatment at 8 day.

**Additional file 5. **Overlap between DEGs and DE miRNA targets during high temperature treatment at 24 day.

**Additional file 6. **Differentially methylated loci.

**Additional file 7. **DMR-associated genes.

**Additional file 8. **Overlap between DEGs and DMR-associated genes during high temperature treatment at 8 day.

**Additional file 9. **Overlap between DEGs and DMR-associated genes during high temperature treatment at 24 day.

## Data Availability

All sequencing data was deposited into the NCBI database under Accession Number SAMN27281867. The data supporting the findings of this study are available from the corresponding authors upon reasonable request.

## Abbreviations

GA: Gibberellin
SAM: Shoot apical meristem
IM: Inflorescence meristem
FT: Flowering locus T
SOC1: Suppressor of overexpression of constans 1
CO: Constans
PIF4: Phytochrome-interacting factor 4
LD: Long-day
AP2: Apetala 2
CAL: Cauliflower
LFY: Leafy
PCA: Principal component analysis
DEG: Differentially expressed gene
GO: Gene ontology
GA20OX1: GA 20-oxidase 1
