# Supplementary material for: Molecular basis of high temperature-induced bolting in lettuce revealed by multi-omics analysis
Source: BMC Genomics. 2022 Aug 12;23:580. doi: 10.1186/s12864-022-08814-z (PMC9373282; doi:10.1186/s12864-022-08814-z)
Supplement: Supplementary file 1 — Additional file 1: Supplementary Fig. 1. Principal component analysis (PCA) of control and high temperature treated samples at different time points. PCA of the 21 RNA-Seq datasets showed 7 distinct groups. RNA-Seq data related to control at 0 day (C0), control at 2 day (C2), control at 8 day (C8), control at 24 day (C24), 2 days post high temperature treatment (HT2), 8 days post high temperature treatment (HT8) and 24 days post high temperature treatment (HT24). Supplementary Fig. 2. GO and KEGG analysis of differentially expressed genes (DEGs) in lettuce tips between HT2 and C2. (a-b) Scatter diagram of GO enrichments of up (a)- and down (b)-DEGs between HT2 and C2. (c-d) Scatter diagram of KEGG enrichments of up (c)- and down (d)-regulated DEGs between HT2 and C2. Gene ratio is the significant DEG number to the background number in a specific pathway. The dot color represents -log10(P), and a higher value indicates greater pathway enrichment. Supplementary Fig. 3. GO and KEGG analysis of DEGs in lettuce tips between HT8 and C8. Scatter diagram of pathway enrichments of up (a, c)- and down (b, d)-regulated DEGs after high temperature treatment at 8 day. Gene ratio is the significant DEG number to the background number in a specific pathway. The dot color represents –log10(P), and the red dot color indicates greater pathway enrichment. Supplementary Fig. 4. GO and KEGG analysis of differentially expressed genes (DEGs) in lettuce tips between HT24 and C24. Scatter diagram of pathway enrichments of up (a, c)- and down (b, d)-regulated DEGs post high temperature treatment at 24 day. Gene ratio is the significant DEG number to the background number in a specific pathway. The red dot color indicates greater pathway enrichment. Supplementary Fig. 5. Gene expression pattern and functional category over the time course during high temperature treatment. The brown color indicated higher expression while blue indicated lower expression. Supplementary Fig. 6. The expression l [file 12864_2022_8814_MOESM1_ESM.docx]

**
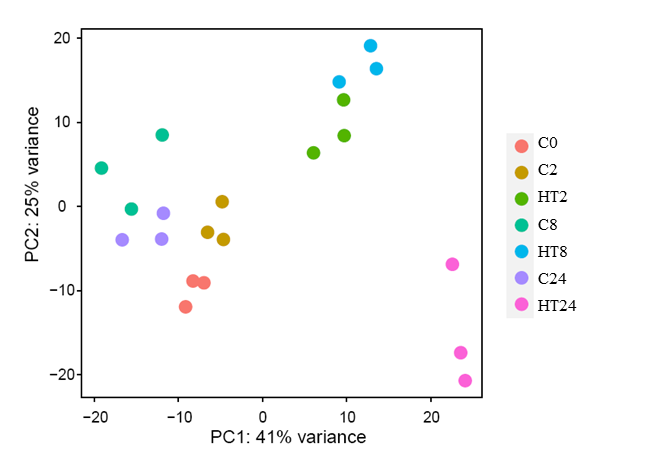
**

**Supplementary Fig. 1.** Principal component analysis (PCA) of control and high temperature treated samples at different time points.

PCA of the 21 RNA-seq datasets showed 7 distinct groups. RNA-seq data related to control at 0 day (C0), control at 2 day (C2), control at 8 day (C8), control at 24 day (C24), 2 days post high temperature treatment (HT2), 8 days post high temperature treatment (HT8) and 24 days post high temperature treatment (HT24).


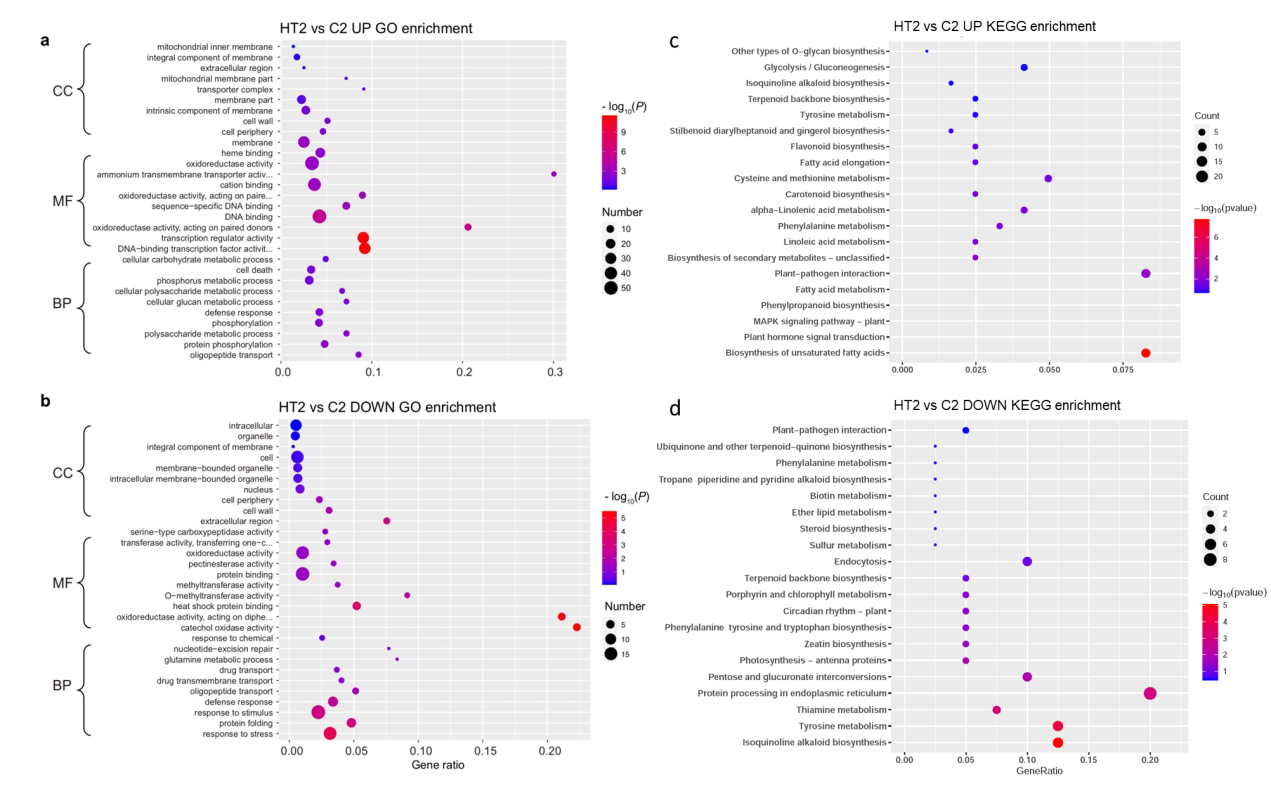


**Supplementary Fig. 2.** GO and KEGG analysis of differentially expressed genes (DEGs) in lettuce tips between HT2 and C2.

(**a**-**b**) Scatter diagram of GO enrichments of up (**a**)- and down (**b**)-DEGs between HT2 and C2. (**c**-**d**) Scatter diagram of KEGG enrichments of up (**c**)- and down (**d**)-regulated DEGs between HT2 and C2. Gene ratio is the significant DEG number to the background number in a specific pathway. The dot color represents -log_10_(*P*), and a higher value indicates greater pathway enrichment.

**
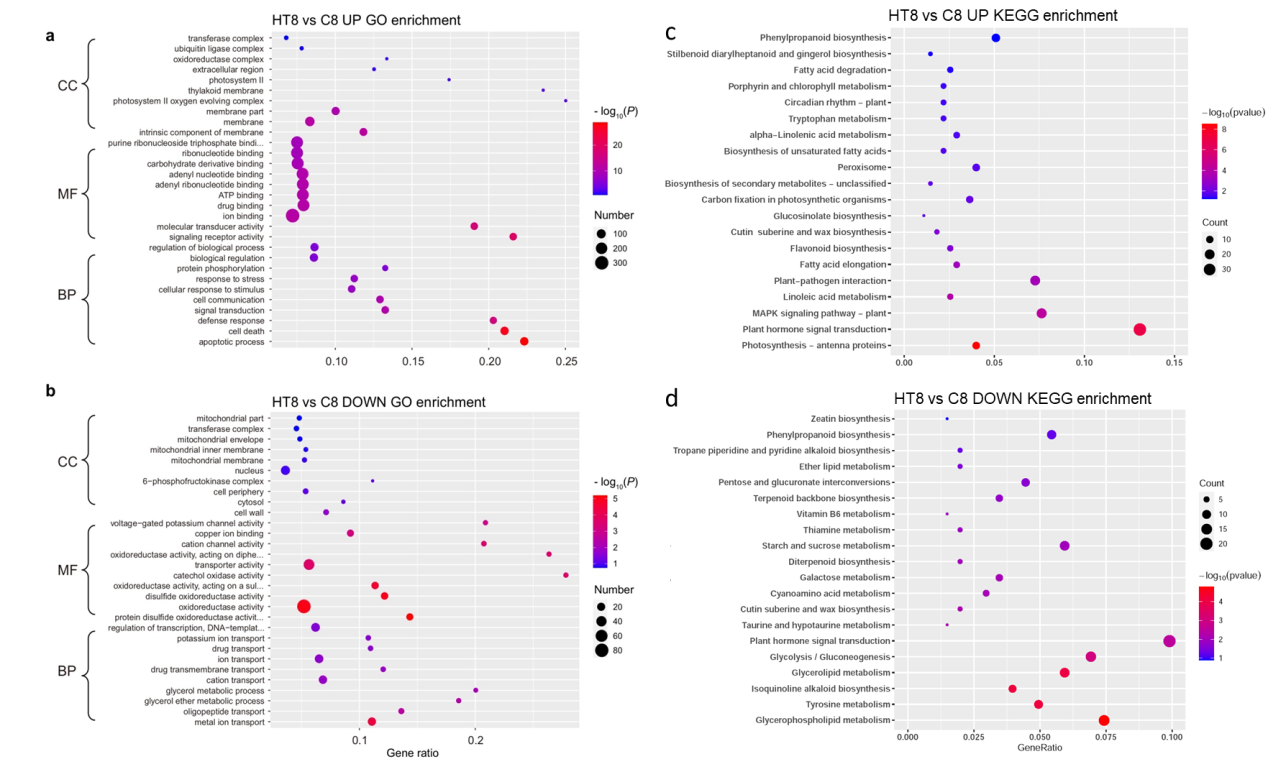
**

**Supplementary Fig. 3.** GO and KEGG analysis of DEGs in lettuce tips between HT8 and C8.

Scatter diagram of pathway enrichments of up (**a, c**)- and down (**b, d**)-regulated DEGs after high temperature treatment at 8 day. Gene ratio is the significant DEG number to the background number in a specific pathway. The dot color represents –log_10_(*P*), and the red dot color indicates greater pathway enrichment.


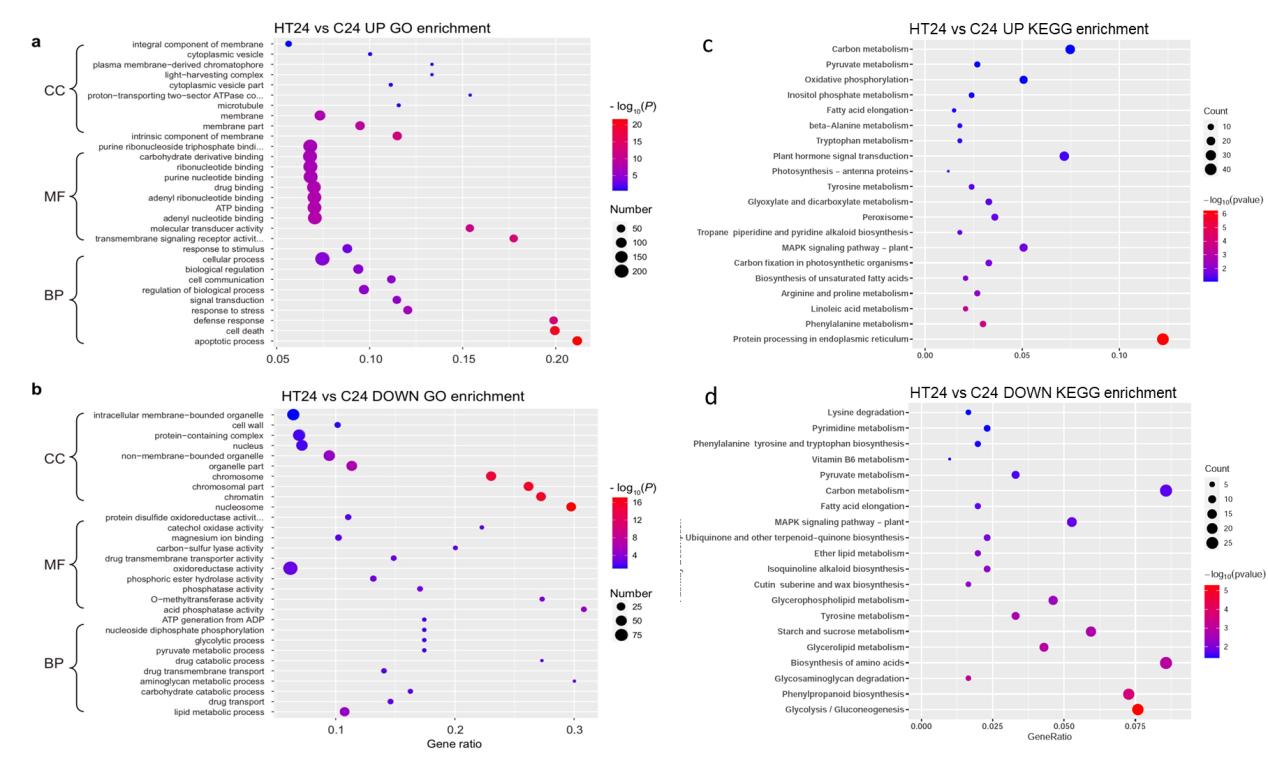


**Supplementary Fig. 4.** GO and KEGG analysis of differentially expressed genes (DEGs) in lettuce tips between HT24 and C24.

Scatter diagram of pathway enrichments of up (**a, c**)- and down (**b, d**)-regulated DEGs post high temperature treatment at 24 day. Gene ratio is the significant DEG number to the background number in a specific pathway. The red dot color indicates greater pathway enrichment.

**
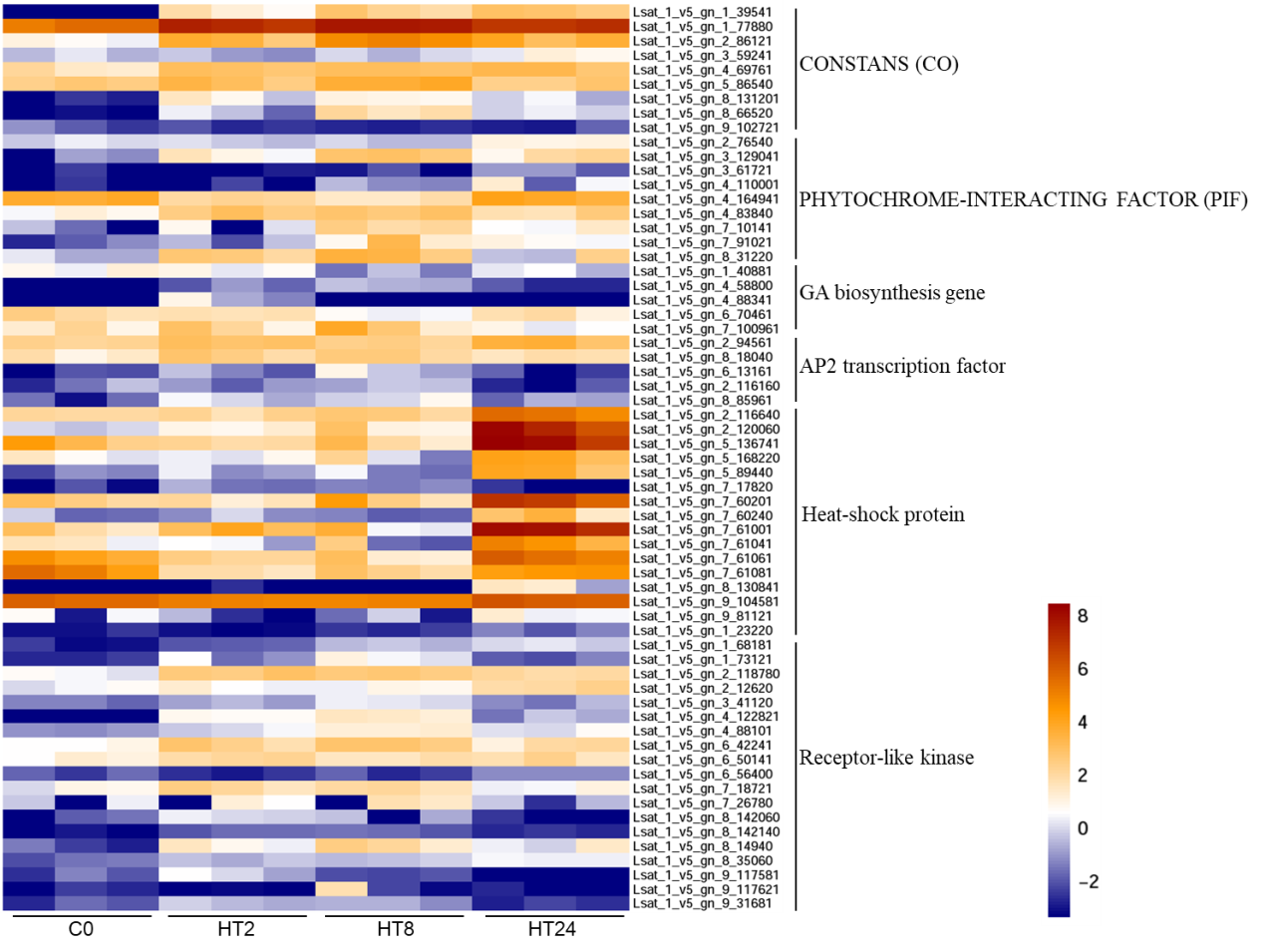
**

**Supplementary Fig. 5.** Gene expression pattern and functional category over the time course during high temperature treatment. The brown color indicated higher expression while blue indicated lower expression.

**
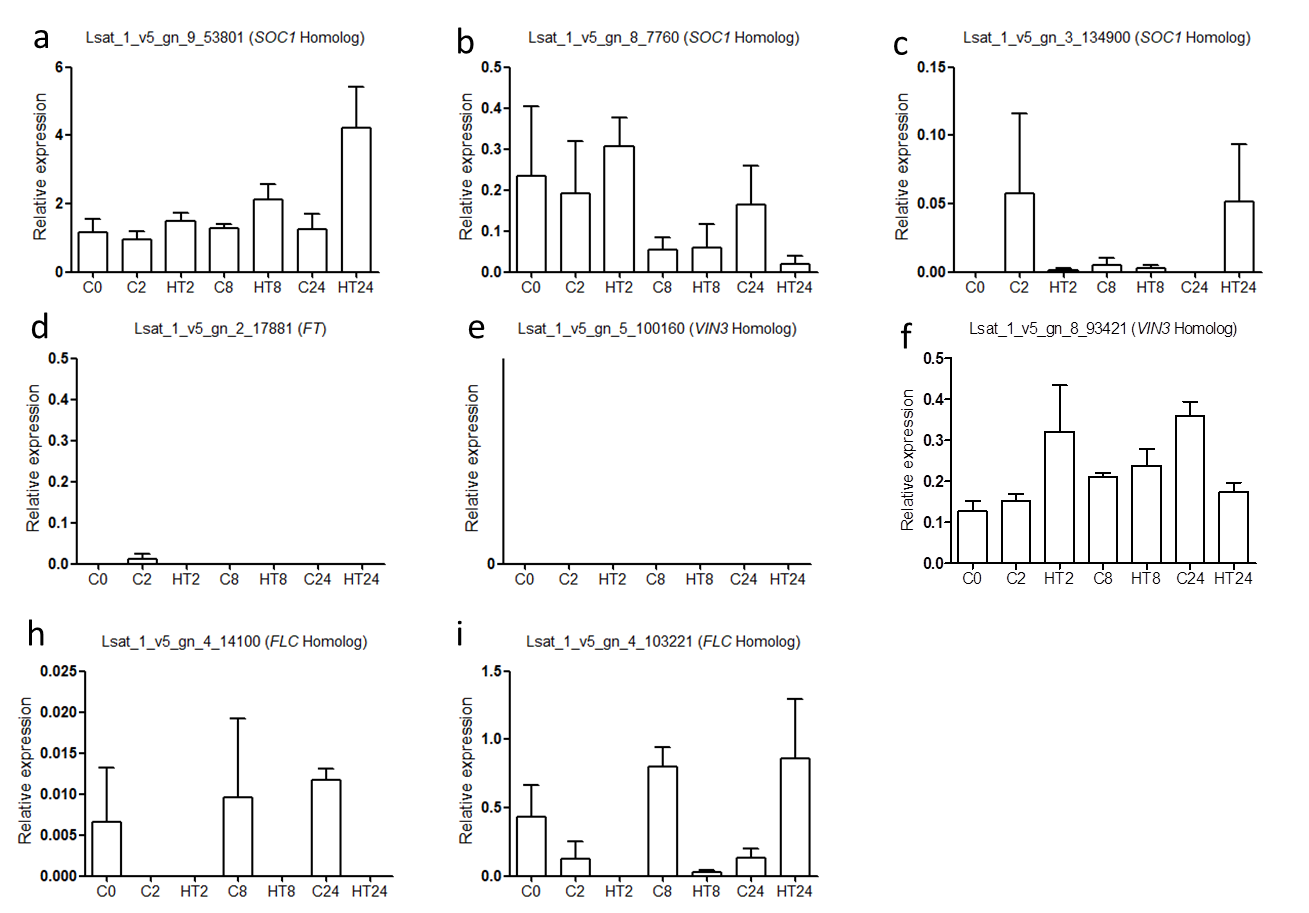
**

**Supplementary Fig. 6.** The expression level of *SOC1*, *FT*, *VIN3* and *FLC* based on the RNA-Seq data.

**
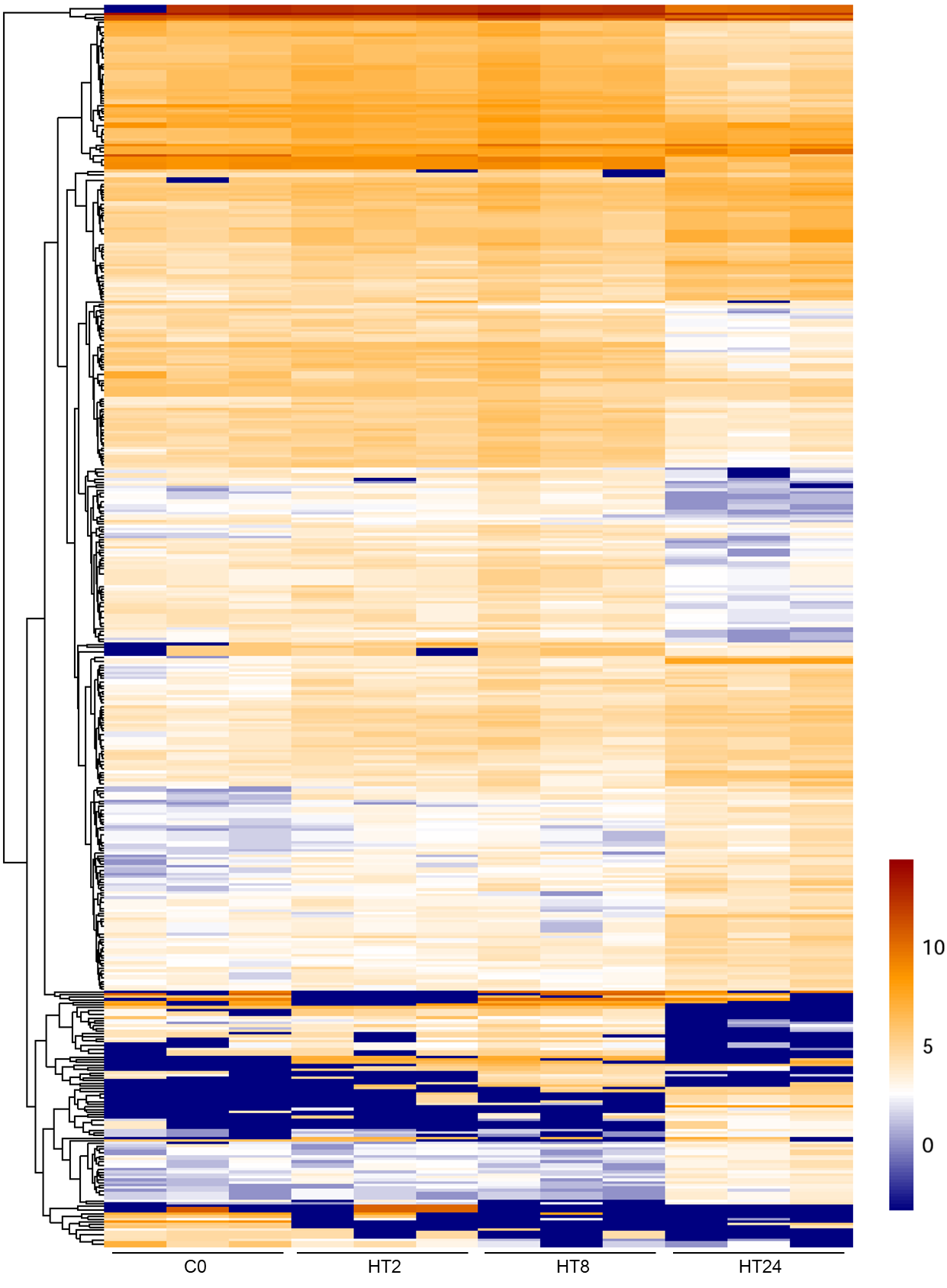
**

**Supplementary Fig. 7.** The expression pattern of miRNAs over the time course during high temperature treatment in lettuce. The brown color indicated higher expression, while blue indicated lower expression.

**.**

**Supplementary Fig. 8.** The distribution patterns of differentially methylated regions (DMRs) on chromosomes in lettuce.
